# ARH Family of ADP-Ribose-Acceptor Hydrolases

**DOI:** 10.3390/cells11233853

**Published:** 2022-11-30

**Authors:** Hiroko Ishiwata-Endo, Jiro Kato, Sachiko Yamashita, Chanbora Chea, Kazushige Koike, Duck-Yeon Lee, Joel Moss

**Affiliations:** 1 Laboratory of Translational Research, Pulmonary Branch, National Heart, Lung, and Blood Institute, National Institutes of Health, Bethesda, MD 20892, USA; 2 Biochemistry Core, National Heart, Lung, and Blood Institute, National Institutes of Health, Bethesda, MD 20892, USA

**Keywords:** ADP-ribosylation, macrodomains, sirtuins, poly(ADP-ribosyl)ation, ADP-ribosyl-acceptor hydrolases (ARHs), tumorigenesis, parthanatos

## Abstract

The ARH family of ADP-ribose-acceptor hydrolases consists of three 39-kDa members (ARH1-3), with similarities in amino acid sequence. ARH1 was identified based on its ability to cleave ADP-ribosyl-arginine synthesized by cholera toxin. Mammalian ADP-ribosyltransferases (ARTCs) mimicked the toxin reaction, with ARTC1 catalyzing the synthesis of ADP-ribosyl-arginine. ADP-ribosylation of arginine was stereospecific, with β-NAD^+^ as substrate and, α-anomeric ADP-ribose-arginine the reaction product. ARH1 hydrolyzed α-ADP-ribose-arginine, in addition to α-NAD^+^ and *O*-acetyl-ADP-ribose. Thus, ADP-ribose attached to oxygen-containing or nitrogen-containing functional groups was a substrate. *Arh1* heterozygous and knockout (KO) mice developed tumors. *Arh1*-KO mice showed decreased cardiac contractility and developed myocardial fibrosis. In addition to *Arh1*-KO mice showed increased ADP-ribosylation of tripartite motif-containing protein 72 (TRIM72), a membrane-repair protein. ARH3 cleaved ADP-ribose from ends of the poly(ADP-ribose) (PAR) chain and released the terminal ADP-ribose attached to (serine)protein. ARH3 also hydrolyzed α-NAD^+^ and *O*-acetyl-ADP-ribose. Incubation of *Arh3*-KO cells with H_2_O_2_ resulted in activation of poly-ADP-ribose polymerase (PARP)-1, followed by increased nuclear PAR, increased cytoplasmic PAR, leading to release of Apoptosis Inducing Factor (AIF) from mitochondria. AIF, following nuclear translocation, stimulated endonucleases, resulting in cell death by Parthanatos. Human *ARH3*-deficiency is autosomal recessive, rare, and characterized by neurodegeneration and early death. *Arh3*-KO mice developed increased brain infarction following ischemia-reperfusion injury, which was reduced by PARP inhibitors. Similarly, PARP inhibitors improved survival of *Arh3*-KO cells treated with H_2_O_2_. ARH2 protein did not show activity in the in vitro assays described above for ARH1 and ARH3. ARH2 has a restricted tissue distribution, with primary involvement of cardiac and skeletal muscle. Overall, the ARH family has unique functions in biological processes and different enzymatic activities.

## 1. Dedication to Elaine and Myron Jacobson

### 1.1. Early Studies on Amino Acid Modification by ADP-Ribose

I (J.M.) have had the privilege of knowing and working with Mike and Elaine Jacobson since the 1970s. Our laboratory’s first collaborative publications with Mike and Elaine Jacobson were published in the 1980s [1,2,3,4,5]. An early research goal of the Jacobson laboratory was to assess the scope of protein modification by ADP-ribose in vivo. This work preceded by many years the development of mass spectral methods that allowed direct detection of amino acids modified with ADP-ribose. The earlier approach utilized by the Jacobsons involved development of chemical methods for the selective release of ADP-ribose from different amino acid acceptors and detection of the released ADP-ribose using fluorescent methods. This powerful, innovative approach led the Jacobsons to identify the presence ADP-ribosylated amino acids in proteins.

By way of background, in 1974, I started as a post-doctoral fellow in the laboratory of Martha Vaughan in the National Heart, Lung, and Blood Institute at the U.S. National Institutes of Health. Dr. Vaughan was studying the mechanism of action of cholera toxin, the protein toxin responsible for the fluid and electrolyte abnormalities characteristic of clinical cholera [6,7]. Dr. Vaughan had data showing that the effects of cholera toxin were due to increased cyclic AMP formation and occurred not only on intestinal epithelial cells, the target of cholera toxin but on other cells, that were unlikely targets of the toxin, such as adipocytes [8]. It was unclear from other laboratories whether NAD^+^ was involved in the toxin-catalyzed reaction. The effects of NAD^+^ as a substrate for other bacterial toxins had been shown by Collier [9], and other workers in the late 1960s [10,11]. Based on the hypothesis that if cholera toxin required NAD^+^ and were an ADP-ribosyltransferase with an unknown amino acid/protein acceptor, later shown to be arginine and a guanine nucleotide-binding protein, respectively, then it might also activate the ADP-ribose-nicotinamide bond of NAD^+^ to promote the transfer, and thus making NAD^+^ susceptible to hydrolysis, generating ADP-ribose and nicotinamide. That indeed was the case [12,13,14,15]. But, if the ADP-ribose-nicotinamide bond were activated, could the hydrolytic reaction use amino acids as a model for the protein acceptor? That too was true and arginine was the acceptor, rather than lysine [16]. Other peptides containing a guanidino group worked as well. Further studies showed that mammalian and avian tissues had enzymes that also catalyzed the ADP-ribosylation of arginine [17,18,19].

In 1985, the Jacobsons’ chemical approach for the identification of modified proteins allowed the detection in vivo of proteins modified at arginine residues and quantification showed that protein modification by ADP-ribose monomers greatly exceeded modification by ADP-ribose polymers [1]. Of note, in 1990, this approach was extended to the detection of plasma membrane proteins modified in vivo at cysteine residues [5]. Pertussis toxin was shown previously to modify a cysteine in another G protein, Gαi [20,21]. The toxins provided insight to what mammalian cells might be doing. The Jacobsons’ novel techniques provided chemical confirmation.

The chemical methods pioneered by the Jacobsons demonstrated that arginine was modified with ADP-ribose and our studies showed that mammalian enzymes could catalyze the ADP-ribosylation of free arginine and arginine in peptides and proteins. These findings led to the question of whether the ADP-ribosylation reaction was reversible. To address this issue, the Jacobsons and our laboratory collaborated on the search for an enzyme that might hydrolyze the ADP-ribose-arginine bond, leading to two collaborative manuscripts on the identification of the activity, subsequently attributed to ADP-ribosyl-arginine hydrolase 1 (ARH1) [2,3]. The Jacobsons clearly took the lead in identifying the potential substrates for mammalian enzymes. Further studies, discussed below, demonstrated the existence of the proteins and enabled the generation of cell and mouse models.

### 1.2. Collaborative Studies with the Jacobsons on Acetal Linkages

In 1995, the Jacobson laboratory published a paper describing the presence of proteins modified in vivo by ADP-ribose monomers with chemical properties characteristic of linkages to hydroxyl groups of serine, threonine or tyrosine residues [22]. Subsequently, the Hottiger and Ahel laboratories confirmed the presence of an ADP-ribose-serine linkage associated with the initiating amino acid, leading to the poly(ADP-ribosyl)ation of protein by PARP-1 [23,24]. The cleavage of the ADP-ribose-serine linkage was catalyzed by another member of the ARH family, ARH3 [23,24].

Although the ARH family members, ARH1, 2, and 3, share some similarities in structure, they are different in the types of reactions that they catalyze, as noted below. ARH3 catalyzes a number of reactions based on its hydrolysis of ADP-ribose-acceptor bond. Further involvement in host metabolic regulation may be discovered as more acceptors are defined.

The studies by the Jacobsons show some of the reaction products that are generated in vivo and what types of ADP-ribosyltransferases and ADP-ribosyl-acceptor hydrolases might exist. Macrodomain-containing proteins also catalyze similar reactions to the ARH proteins but the substrate specificities and the reaction conditions appear to differ [25,26,27]. There are many macrodomains, from viruses to bacteria to mammals, and others might be discovered that have properties more similar to the ARHs [28].

### 1.3. Regarding the Path to Dermatology Products

The Jacobsons have used their basic interest in NAD^+^ metabolism to translate their studies from the laboratory into the clinic. At present, the importance of ADP-ribosylation in human disease from cancers (e.g., PARP inhibitors) [29,30,31] to neurodegeneration (e.g., ARH3 deficiency) [32,33,34,35,36,37,38] has been established in a number of laboratories. These findings stem from many of the initial observations by the Jacobsons’ laboratory. The Jacobsons have had their major clinical impact in dermatology, where the impact of ADP-ribosylation on disease pathogenesis and the potential for therapy were demonstrated.

A 1979 paper from the Jacobson laboratory [39] first demonstrated simultaneous stimulation of ADP-ribose polymer formation and NAD^+^ depletion in cells following genotoxic stress. This finding stimulated a long-term interest in NAD^+^ metabolism in tissues with sub-optimal vitamin B3 status and/or conditions resulting from excess or chronic genotoxic stress. Sun-exposed skin became a major focus of these studies, including an interest in progression to skin cancers. These observations led to the discovery that topical application of specific lipophilic derivatives of nicotinic acid could promote selective enhancement of skin NAD^+^ content. Dermatology products containing this technology were shown to promote epidermal differentiation and thus promote skin barrier function in photo-aged skin [40] and prevent barrier impairment in retinoid-treated skin [41]. These studies also led to the discovery and characterization of an epidermal G-protein coupled nicotinic acid receptor that stimulates epidermal differentiation, and that this receptor is non-functional in squamous cells from skin tumors [42]. The ability of this technology to promote skin barrier repair has wide therapeutic implications since skin barrier impairment is a cause or contributor to numerous dermatological conditions and diseases with a wide range of severity across the human life span. Dermatology products based on this technology are now widely marketed.

## 2. Introduction

Cholera toxin is the bacterial product synthesized by *Vibrio cholerae*, which is responsible for the fluid and electrolyte flux characteristic of Cholera [6]. It is an AB toxin, with the A subunit responsible for catalytic activity, while the 5 B subunits exist as a complex that binds the cell surface receptor, ganglioside GM1, and promotes the entry of the A subunit [43,44]. Our first studies on the cholera toxin A subunit to help define its function showed that it was an NAD^+^ glycohydrolase, acting in vitro independent of the B oligomer [12]. Under the assumption that the NAD^+^ glycohydrolase resulted from activation of the ADP-ribose-nicotinamide bond of NAD^+^, we examined if free amino acids or related compounds could serve as ADP-ribose acceptors [16]. Subsequent studies showed that arginine, guanidine and molecules containing a guanidino functional group (e.g., agmatine), served as ADP-ribose acceptors, whereas lysine and other amino acids were inactive [16]. Subsequent studies from several laboratories identified the acceptor protein in the host cell as a guanine nucleotide-binding protein, termed Gαs, which activates the adenylyl cyclase catalytic unit [13,14,15]. Further studies on cholera toxin activity demonstrated that the cholera toxin A subunit ADP-ribosyltransferase activity was stimulated by another guanine nucleotide-binding protein, which was active with GTP bound and inactive with bound GDP. This 21-kDa protein, termed ADP-ribosylation factor or ARF was shown to be an allosteric activator of the cholera toxin A subunit ADP-ribosyltransferase activity [16,45,46,47].

The fact that cholera toxin was an ADP-ribosyltransferase led to the question of whether it was mimicking the action of mammalian transferases and led to the identification of avian and mammalian NAD^+^:arginine ADP-ribosyltransferases [48]. The mammalian ADP-ribosyltransferase family was shown to consist of five ARTCs with sequence similarities to cholera toxin [49]. ARTC1 was similar in catalytic properties to cholera toxin A subunit; it used free arginine as an ADP-ribose-acceptor. Both enzymes catalyzed stereospecific S_N_2-like reactions, with β-NAD^+^ serving as substrate and the reaction product being α-anomeric ADP-ribose-arginine [48]. The site of modification is the guanidino functional group of arginine, with ADP-ribose attached to arginine through its C-1” position. Other basic amino acids (e.g., lysine) could not be ADP-ribosylated by either the toxin or the mammalian equivalents (e.g., ARTC1) [16]. ADP-ribosylome of endogenous ADP-ribosylation sites identified arginine (86%) as a major amino acid to be ADP-ribosylated in mouse liver [50].

ARTC1 is an exoenzyme, anchored to the cell surface through a glycosylphosphatidylinositol (GPI) anchor [51]. Its amino acid sequence contains regions compatible with other transferase catalytic sites. ARTC2 is found in rodents, resulting in the murine enzyme exhibiting properties of an NAD^+^:arginine ADP-ribosyltransferase [52], whereas the rat enzyme is an NAD^+^ glycohydrolase [53]. Human ARTC2 is a pseudogene [54]. The ability of the mouse and rat enzymes to use arginine as an acceptor as opposed to hydrolyzing NAD^+^ to ADP-ribose and nicotinamide is based on minor changes in amino acid sequence at the catalytic site, with the murine enzyme being an ADP-ribosyltransferase and the rat enzyme being an NAD^+^ glycohydrolase [55].

ARTC3 and ARTC4 have not been shown to be NAD^+^:arginine ADP-ribosyltransferases [49]. Both appear to be cell surface enzymes. ARTC5 differs in sequence at its carboxy end from the other ARTCs, in that it no longer has the signal sequence needed to add a GPI anchor [56]. It does, however, have the amino terminal sequence needed to export the protein, similar to the other ARTC protein family members that are GPI linked. Thus, ARTC5 appears to be secreted without the GPI-tether to keep it linked to the cell membrane. Similar to ARTC1, ARTC5 also catalyzes the ADP-ribosylation of arginine, however, ARTC5 appears to be primarily an NAD^+^ glycohydrolase [57]. In studies of substrates of wild-type and *Artc1*-deficient mouse skeletal muscle and heart, the Hottiger laboratory observed that most of the ADP-ribosylated substrates found in WT skeletal muscle and heart were not observed in *Artc1*-deficient mice. These data suggest that in mouse skeletal muscle and heart, the primary enzyme responsible for ADP-ribosylation of arginine in skeletal muscle and heart is ARTC1 [58].

## 3. ADP-Ribose-Acceptor Hydrolase (ARH) Overview

Cholera toxin is presumably toxic since its ADP-ribosylation results in the uncontrolled activation of Gαs [44]. If this were the case, why aren’t the mammalian and avian ADP-ribosyltransferases toxic? We postulated that ADP-ribosylation levels could be controlled by enzymes that cleave the ADP-ribose-arginine linkage, generating free (arginine)protein, thus completing a partial-ADP-ribosylation cycle (nicotinamide is released in each round of the cycle). A search for an ADP-ribose-arginine cleavage enzyme led to the discovery of ARH1 [3].

**Figure 1:**
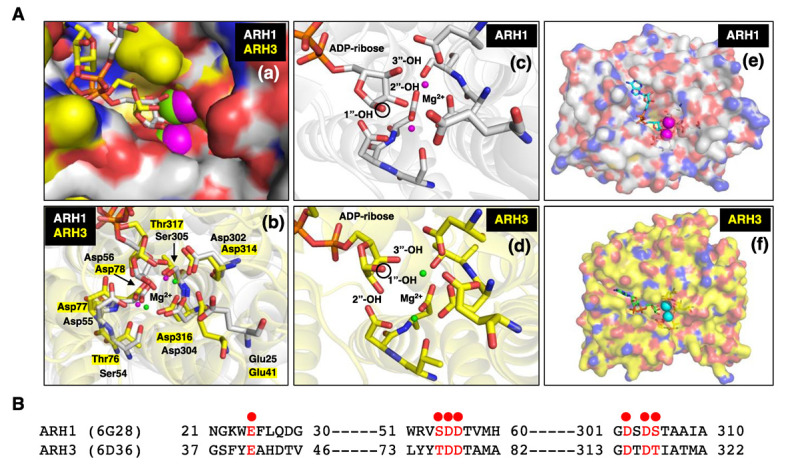
(**A**) (**a**) Close-up surface representation of human ARH1 (white, PDB ID: 6g28) [98] and ARH3 (yellow, PDB ID: 6d36) [99] crystal structures with electro-negative (red) and -positive atoms (blue). The binding pocket of ADP-ribose on surface of ARH1 and ARH3 is near the bound Mg^2+^ ions (magenta-ARH1, green-ARH3) [23,76,98,99,100]. (**b**) Crystal structure of ARH1 and ARH3 with ADP-ribose and Mg^2+^ ions (ARH1-magenta, ARH3-green). Glu25, Ser54, Asp55, Asp56, Asp302, Asp304, and Ser305 in ARH1, and Glu41, Thr76, Asp77, Asp78, Asp314, Asp316, and Thr317 in ARH3 (yellow highlight) hydrogen-bonding amino residues near Mg^2+^ are critical for binding of ADP-ribose, and ARH1 and ARH3 hydrolase activities. (**c**,**d**) Close-up two Mg^2+^ ions and ADP-ribose in binding pocket of ARH1 (**c**) and ARH3 (**d**). The black circle on ribose indicates the 1”-OH of ribose that is an attachment site of arginine or serine residue of modified protein, *O*-acetyl or nicotinamide of α-NAD^+^ [28]. (**e**,**f**) Surface representation of human ARH1 (white, PDB ID: 6g28) and ARH3 (yellow, PDB ID: 6d36) crystal structures. ADP-ribose (cyan-ARH1, green-ARH3) and Mg^2+^ ions (magenta-ARH1, cyan-ARH3) are bound in the pocket of ARH1 or ARH3. (**B**) Structure-guided alignment of selected ARH1 and ARH3 protein residues (red dot) bound with Mg^2+^ ions. Crystal structures of ARH1 (PDB ID: 6g28) and ARH3 (PDB ID: 6d36) with bound ADP-ribose and magnesium were taken from the Protein Data Bank (https://www.rcsb.org, accessed on 13 October 2022). Figures and structure analysis were created with Pymol (http://pymolorg, accessed on 13 October 2022).

**Figure 2:**
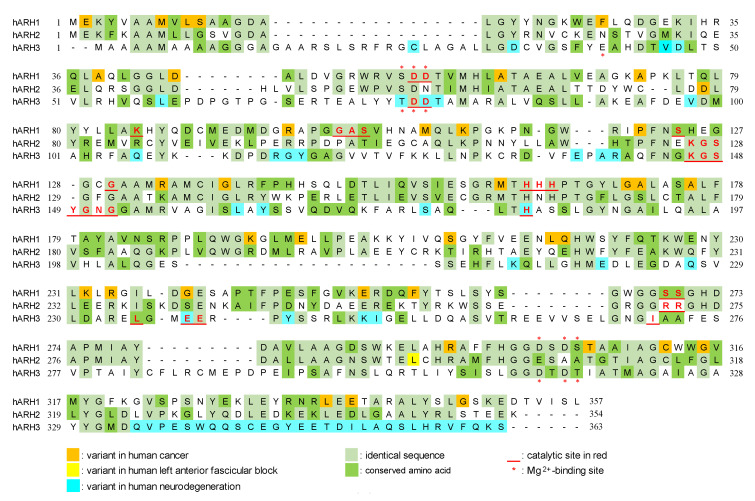
Modified figure of Oka et al. [59]. Alignment of amino acid sequences of human ARH1 (P54922), ARH2 (Q8NDY3), and ARH3 (Q9NX46). Sequence alignment was performed by T-Coffee method [59,101]. Mg^2+^-binding sites and critical amino acids for ARH1 [77,98,102], ARH2 [84], and ARH3 [24,99,100,103,104,105] activity were based on experiments and predictions. Variants in human cancer [77], left anterior fascicular block [84], and neurodegeneration [32,33,34,35,36,37,38] are based on previous reports and the Genome Aggregation Database (https://gnomad.broadinstitute.org/, accessed on 13 October 2022). Amino acid sequences were obtained from UniProt (https://www.uniprot.org/, accessed on 13 October 2022).

**Figure 3:**
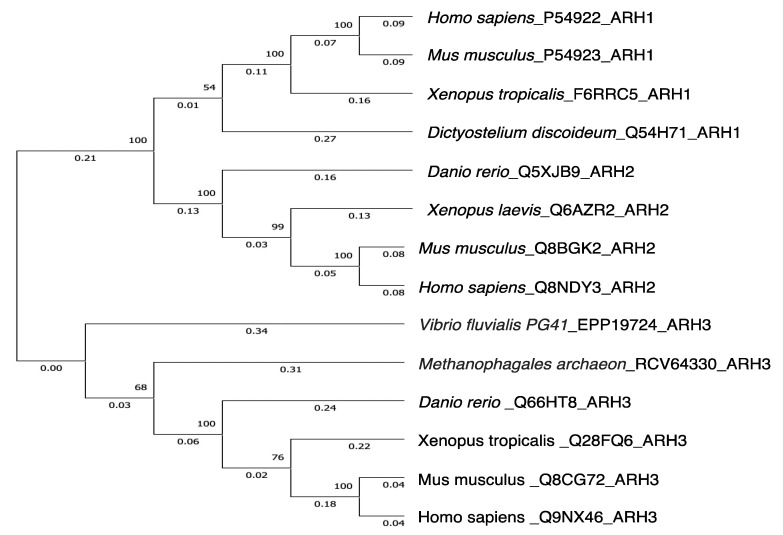
Phylogenetic trees showing genetic relationships of ARH1, ARH2 and ARH3.The tree was constructed by the UPGMA in the Evolutionary Genetics Analysis X (MEGAX) program (https://www.megasoftware.net/, accessed on 13 October 2022). The evolutionary distance was computed using p-distance method by the MEGAX [106,107]. Reference sequences are indicated by species_accession number_protein name. The bootstrap values (%) of 500 replications are shown at each internal node. Genetic variations between human ARH1 and human ARH2 or human ARH3 were 0.57 and 0.82, respectively. Amino acid sequences were obtained from UniProt (https://www.uniprot.org/, accessed on 13 October 2022).

**Figure 4:**
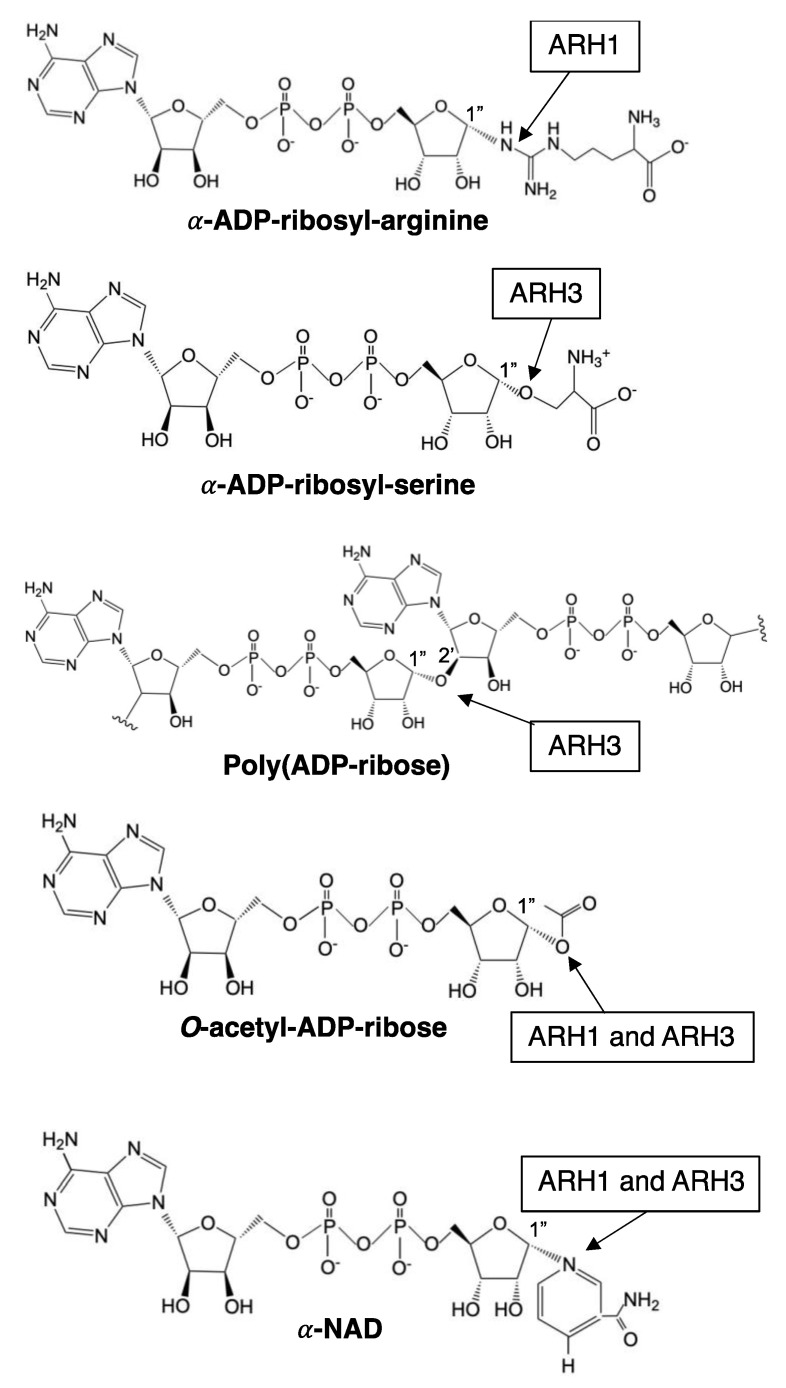
Schematic diagram of substrates of ARH1 and ARH3. Arrow indicates linkage cleaved by ARH1 and ARH3. ARH1 and/or ARH3 catalyze stereospecific hydrolysis at α-ADP-ribosyl-arginine (ARH1) [72,73], α-ADP-ribosyl-serine (ARH3) [23,24], poly(ADP-ribose) (ARH3) [59], *O*-acetyl-ADP-ribose (ARH1 and ARH3) [70,82], and α-NAD^+^ (ARH1 and ARH3) [76]. ChemDraw from PerkinElmer informatics was used to draw the diagram.

Genomic analysis showed that ARH1 is part of an ARH family composed of three family members, ARH1, 2 and 3 [59]. The ARHs share similarities/identities, structure and phylogenetic origins (Figure 1, Figure 2 and Figure 3), but the ARHs differ considerably in their enzymatic properties (Figure 4). Properties of ARHs are summarized in Table 1. Different model reactions were designed to identify the catalytic properties of the ARHs. ADP-ribose is linked to a number of different acceptors by different families of transferases. PARP family members can ADP-ribosylate various amino acids (e.g., aspartate/glutamate [60,61], serine [23], lysine [62]), as well as synthesize long-chain, branched poly(ADP-ribose) [60,62,63]. Sirtuins can serve as ADP-ribosyltransferases, as well as catalyze the formation of *O*-acetyl-ADP-ribose, through an NAD^+^-dependent, de-acetylation reaction [64,65]. Their individual properties will be discussed below. ARTC1 and ARTC5 can ADP-ribosylate arginine, using β-NAD^+^ as substrate, forming the α-anomeric product. CD38 and related enzymes catalyze the formation of cyclic ADP-ribose, while retaining the ability to hydrolyze the compound, releasing ADP-ribose [66,67]. In vivo, it is postulated that α-NAD^+^ can form, perhaps as a side product of a redox reaction involving β-NAD^+^ [68,69]. The role of α-NAD^+^, or its possible action as a toxic metabolite that needs to be destroyed has not been determined. Overall, the transferases appear to be stereospecific in the use of β-NAD^+^, with the formation of an α-anomeric product [48]. ARH1 and ARH3 appear to hydrolyze the α-anomeric product, as might be expected if the transferases and ARHs were opposing arms of ADP-ribosylation cycles. Thus far, ARH2 has not been shown to have enzymatic activities [59,70]. The ARHs, however, show considerable specificity in their choice of substrate and in their relative hydrolytic activities.

Some of macrodomain-containing proteins appear to share the ability with the ARHs to hydrolyze the ADP-ribose-acceptor linkages [28]. They are found from viruses to bacteria to mammalian species. However, the macrodomains appear to differ in their catalytic properties and in the structure of the ADP-ribose binding site [28].

The ARHs have also been studied with the aid of murine models, in an effort to understand the extent of organ involvement in knockout and heterozygote animals and potential for re-purposing drugs for treatment of human deficiency states.

## 4. ARH1

As noted above, the studies on cholera toxin ADP-ribosyltransferase activity led to the observation that the toxin used arginine and β-NAD^+^ as substrates and catalyzed the stereospecific formation of α-ADP-ribose-arginine with release of nicotinamide [71]. The reaction product could then be cleaved by a soluble protein found in many mammalian cells to yield arginine and ADP-ribose [72,73]. The responsible protein, termed ARH1 for ADP-ribose-acceptor-hydrolase-1, was purified, cloned and a knockout mouse was generated.

Enzyme properties: ARH1 is a 39-kDa protein, with enzymatic and structural equivalents found in human and rodents [72,74]. In support of its serving as an opposing arm of the cholera toxin- and ARTC1-catalyzed ADP-ribosyltransferase pathways, ARH1 cleaved the stereospecific product of the ARTC1 and cholera toxin β-NAD^+^:arginine ADP-ribosyltransferase-catalyzed reaction [71,75], α-ADP-ribosyl-arginine, generating ADP-ribose and arginine [73]. The β-anomer of ADP-ribosylarginine was not a substrate [73]. The guanidino group appeared to be unaltered by the cycle and, when isolated, could be ADP-ribosylated again [3]. In addition to ADP-ribose-arginine, α-NAD^+^, but not β-NAD^+^, served as a substrate for ARH1, generating ADP-ribose and nicotinamide as products, consistent with the stereospecificity observed earlier with α-ADP-ribosylarginine hydrolysis [76].

Mouse phenotype: An *Arh*1-knockout (KO) mouse was viable but had significant phenotypes depending, in some cases, on age and gender. The *Arh1*-KO and heterozygous mouse developed tumors in multiple organs, with more frequent metastasis [77]. The heterozygous mouse showed tumors that exhibited mutations in the remaining *Arh1* allele that altered its enzymatic activity, so that it was not effective as an ADP-ribosyl-arginine hydrolase, thus the tumor was acting as an *Arh1* KO. Although the WT mouse showed more tumors in male mice, the *Arh1*-KO and heterozygous female mice developed more localized and metastatic tumors [77,78].

Male *Arh1*-KO mice developed a cardiomyopathy, with decreased cardiac contractility noted by MRI and echocardiography [19]. *Arh1*-KO mice also showed a greater susceptibility to cholera toxin activity, with enhanced ADP-ribosylation of the guanine nucleotide-binding protein responsible for the activation of adenylyl cyclase, Gαs, and the pathological response on the intestinal mucosa with increased fluid and electrolyte flux [79,80].

In membrane repair of the myocardium, it appears that there is an ADP-ribosylation cycle, composed of ARTC1, catalyzing the ADP-ribosylation of arginines on the known membrane-repair protein tripartite motif-containing protein 72 (TRIM72) with ARH1 catalyzing the release of the ADP-ribose, thereby completing the cycle. Both ARTC1 and ARH1 are required for effective formation and activity of a TRIM72-mediated, membrane-repair complex [19].

However, of note, ARTC1 appears to act in some situations in the absence of ARH1. In the lung airway, some diseases are characterized by the release α-defensin, i.e., human neutrophil peptide-1 (HNP-1), included among these are idiopathic pulmonary fibrosis (IPF), and asthma, as well as smokers [18,81]. ARTC1 is glycosylphosphatidylinositol (GPI)-anchored, found on the surface of inflammatory cells and airway epithelial cells, and hence, exposed to the inflamed airway. When HNP-1 was isolated from patients, it appears to be ADP-ribosylated in 1 or possibly two sites (arginines 14 and 24). The modified HNP-1 appears to differ in pharmacological properties [81]. In the absence of ARH1 in the airway, the ADP-ribosylated (arginine) HNP-1 undergoes non-enzymatic degradation of the ADP-ribosylated arginine, in a reaction also seen with the model substrate, ADP-ribose-arginine, leading to formation of ornithine, identified by amino acid analysis and mass spectrometry, with release of a compound similar to ADP-ribose-carbodiimide. Given the instability of carbodiimide, it is unlikely that the compound exists in vivo. ARH1 also hydrolyzes poly(ADP-ribose), cleaving ADP-ribose-ADP-ribose linkages [59]. *O*-acetyl-ADP-ribose is also hydrolyzed by ARH1 [70]. As shown for ARH3, the reaction involves release of the acetyl group from the ADP-ribose-C-1” position [76,82]. Thus, ARH1 hydrolyzes ADP-ribose-acceptors with acceptors being O-containing functional groups as well as N-containing functional groups. The ARH1-catalyzed reactions are stimulated by Mg^2+^ [74].

## 5. ARH2

Enzyme properties: ARH2 (so far) does not exhibit any enzymatic activities [70], consistent with differences in primary sequences from critical residues found in ARH1 (e.g., 54-**SDD**T-57, 302-**D**S**DS**-305) and ARH3 (e.g., 76-**TDD**T-79, 313-**D**T**DT**-316) (Mg^2+^-binding sites are indicated in bold). ARH2 is a cytoplasmic protein expressed primarily in heart and skeletal muscle and may be involved in cardiac myofibril assembly and cardiac chamber outgrowth [83].

Mouse phenotype: Thus far, an *Arh2*-deficient mouse model has not been reported. To better understand the role of ARH2, a transgenic mouse needs to be generated.

ARH2 involvement in human disease: Whole-genome sequencing revealed that the *ARH2/ADPRHL1* missense variant p.Leu294Arg in *ARH2/ADPRHL1* is associated with left anterior fascicular block (LAFB) using 405,732 electrocardiograms from 81,192 Icelanders [84]. Proteomics data analysis of mouse brain identified ARH2 in a macromolecule containing synemin, desmin, and triadin, suggesting that ARH2 may associate with other proteins in neurological disorders [85]. *ARH2* appears to be involved in tumorgenicity of uveal melanoma and prostate cancer and act as a tumor suppressor [86,87]. *ARH2* mutation at c.A233T (p.Asp78Val) in prostate cancer cell line, AA/PC, increased colony formation with increased PARP1 protein levels and PAR accumulation [87]. Increased cell proliferation and PARP1 protein levels associated with *ARH2* mutation, D78V, were inhibited by PARP inhibitor olaparib treatment, suggesting that ARH2 mediated a PARP1-dependent cell death pathway [87].

## 6. ARH3

Enzyme properties: ARH3 exhibits significant differences in enzymatic activities from those of ARH1. Please note that both ARHs shared some activities when protein concentrations and times of incubation were varied.

Although PARG is the primary enzyme degrading ADP-ribose polymers, catalyzing both endo-and exo-glycosidic hydrolysis [88,89], ARH3 catalyzed the exocidic hydrolysis of poly(ADP-ribose), generating free ADP-ribose and a shortened oligo-ADP-ribose. The attachment of ADP-ribose in the polymer appears to be through an alpha linkage, thus the cleavage is stereospecific. ARH3 also catalyzes the hydrolysis of the ADP-ribose-serine linkage, whose synthesis was stimulated by histone PARylation factor 1 (HPF1) [90], the initial site of attachment of the poly(ADP-ribose) to the protein acceptor [23,24,91].

*O*-acetyl-ADP-ribose was also a substrate [82]. The acetyl group was observed to migrate among the C-3′′, C-2′′, and C-1′′ positions of the ADP-ribose molecule, in a pH-dependent manner. The site of hydrolysis by ARH3 of the acetyl linkage to ADP-ribose was determined, using H_2_^18^O, to be at the C-1′′ position. The ARH3-catalyzed reactions were stimulated significantly by Mg^2+^ and enhanced somewhat by dithiothreitol [70,82]. Of interest, the macrodomain proteins did not exhibit the same dependence on Mg^2+^ for activity as seen with ARH1 and ARH3 [76].

ARH3 also hydrolyzed α-NAD^+^, but not β-NAD^+^, similar to the findings with ARH1, consistent with the stereospecificity at the C-1′′ of ADP-ribose [76]. In catalyzing this reaction, ARH3 shows significantly more activity than does ARH1. ARH3 also hydrolyzed ADP-ribose-arginine, but at much higher concentrations of enzyme and time of incubation than were needed with ARH1. Thus, as with ARH1, ARH3 hydrolyzes ADP-ribose linked to O- (e.g., ADP-ribose-serine, poly(ADP-ribose)) and N- (e.g., α-NAD^+^) functional groups.

*Arh3*-deficient cells: *Arh3*-deficient cells are susceptible to H_2_O_2_-induced cell death in a poly(ADP-ribose) polymerase (PARP)-1-dependent pathway known as Parthanatos [92,93]. Poly(ADP-ribosylation) of PARP-1 requires histone PARylation factor 1 (HPF1) to transfer the initial ADP-ribose on serine residue of PARP-1 [90]. ARH3 has been shown to hydrolyze the ADP-ribose-serine linkage [23,24]. Human osteosarcoma U2OS cells lacking either HPF1 or ARH3 showed opposite responses upon H_2_O_2_-induced oxidative stress, i.e., *ARH3*-knockout cells accumulated ADP-ribosylated serine, whereas *HPF1*-knockout cells showed decreased serine ADP-ribosylation levels [91]. These findings suggest that ARH3 and HPF1 are primarily modulators of serine ADP-ribosylation. PARP is the primary generator of poly(ADP-ribose) (PAR) and acceptor of PAR in response to DNA damage and PARP-1 activation [88,94]. Degradation of PAR is catalyzed by PAR glycohydrolase (PARG) [95] and ARH3 [93,96]. In *Arh3*-deficient mouse embryonic fibroblasts (MEFs), exposure to H_2_O_2_ results in increased levels of PAR in the nucleus and cytoplasm compared to cells expressing wild-type *Arh3*. Cytoplasmic PAR associates with mitochondrial apoptosis-inducing factor (AIF) resulting in translocation of a cleaved AIF to the nucleus through its nuclear translocation signal (NLS). AIF then activates endonucleases, leading to large-scale DNA fragmentation and cell death [93]. PAR also appears to be involved in regulating endocytosis, a pathway used for the intake of nutrients. The data show that PAR interacts with Rab5, a guanine nucleotide-binding protein associated with plasma membrane and early endosomes, inhibiting its activation and the ability of Rab5 to recruit effectors, resulting in its dissociation from membranes and inhibition of nutrient uptake in MEFs [97]. These and other effects of PAR are responsible for a cell death pathway termed Parthanatos [92].

Mouse phenotype: To better understand the pathophysiological function of *Arh3,* knockout mice were generated. *Arh3*-KO mice were viable but were sensitive to oxidative stress, induced by cerebral ischemia-reperfusion injury. The extent of injury in *Arh3*-KO mice was greater than that seen with their wild-type counterparts [33]. Analysis of the cortical neurons showed enhanced PAR. AIF was localized in nuclei, as expected following release from mitochondria due to increased cytoplasmic PAR. Treatment with Veliparib, a PARP inhibitor, resulted in a normalization of PAR and AIF, suggesting that re-purposing the drug may have a beneficial effect in human disease [33].

ARH3 involvement in human disease: *ARH3* deficiency appears to be responsible for human disease, resulting in an autosomal recessive, neurodegenerative phenotype. Data have been published from several centers on the human phenotype associated with *ARH3* deficiency [32,33,34,35,36,37,38]. The course of the disease is quite variable, although affected individuals may show early death. In the case seen here, neurons of the deceased sibling exhibited Parthanatos. An older sibling showed a behavioral phenotype but was alive into her twenties. Based on the mouse and cell data, it appears that PARP inhibitors may lead to a treatment of this disease.

## 7. Conclusions

The ARH proteins, although similar in size and primary sequence, differ in catalytic activities and proposed function in cells based as well on their intracellular and organ-specific locations. Animal models showed gender (e.g., cardiac contractility), and age (e.g., cardiac fibrosis, heart chamber development, specific responses in addition to organ specificity). Studies involving ARH proteins may contribute to finding targeted therapies for cardiac failure. ARH1 and ARH3 hydrolyze, respectively, arginine- and serine-ADP-ribose linkage. However, ARH1 and ARH3 have the ability to catalyze similar reactions controlling cellular ADP-ribosylation levels, but the rates of reaction may differ. Excessive ADP-ribosylation is associated with human disease as is seen in *ARH3* deficiency. Therefore, hydrolysis of mono-ADP-ribosylated substrates leading to recovery of reusable unmodified proteins is an important process to decrease ADP-ribosylation levels elevated by oxidative stress. PARP inhibitor prevented accumulation of cellular ADP-ribose and cell death upon H_2_O_2_ exposure in *ARH3*-deficient cells, suggesting that PARP is a therapeutic target for the *ARH3*-dependent neurodegenerative diseases. ARH2 does not appear to be responsible for hydrolysis of ADP-ribose-acceptor linkage. *ARH2* deficiency has not been shown to be responsible for human pathology. However, the lack of ARH2 catalytic activity may be the result of the use of model substrates. That may change when the actual substrates of some of the ADP-ribosyl acceptors are tested. Function of ARH2 would be an interesting area of future study.

**Table 1:** ADP-ribose-acceptor hydrolase family.

ARH Family		ARH1	ARH2	ARH3
Length (Accession number)		357 aa (P54922)	354 aa (Q8NDY3)	363 aa (Q9NX46)
Identity/similarity of amino acid sequences to ARH1			47/68% ^a^	22/41% ^a^
Subcellular location		cytoplasm ^b^	cytoplasm ^c^	cytoplasm (65%) mitochondria (25%) nucleus (10%) ^d^
Protein expression		ubiquitous ^e^	muscle ^f^, prostate ^z^, brain ^g^	ubiquitous ^e^
Substrate	α-NAD^+^	+ ^h^	—	+++ ^h^
	β-NAD^+^	no ^h^	—	no ^h^
	*O*AADPr	+ ^i^	—	+++ ^i^
	MAR acceptor	ADPr-arginine ^j^	no ^a^	ADPr-serine ^k^
	PAR acceptor	no ^l^	no ^l^	yes ^l^
	DNA	no ^m^	no ^m^	yes ^m^
	RNA	no ^n^	no ^n^	yes ^n^
Physiological function		bacterial infection ^o^ tumorigenesis ^r^ membrane repair ^t^	cardiac development ^p^	oxidative stress ^q^ DNA/RNA repair ^s^
Disease		cholera ^o^ lung adenocarcinoma ^r^ ovarian cancer ^u^ hyperlipoproteinemia ^x^	uveal melanoma ^v^ cardiac disease ^y^ prostate cancer ^z^	neurodegeneration ^w^

*O*AADPr, *O*-acetyl-ADP-ribose; MARylation, mono-ADP-ribose-acceptor; PARylation, poly-ADP- ribose-acceptor. Presence of activity was indicated by “no” or “yes”. “+” and “+++” indicate ARH3 has higher activity than ARH1 for the respective substrate. “—” indicates that no data have been reported for those activities. Table is based on reports, ^a^ [59], ^b^ [72], ^c^ [108], ^d^ [93], ^e^ [109], ^f^ [83], ^g^ [85], ^h^ [76], ^i^ [70], ^j^ [19,73,79], ^k^ [23], ^l^ [59], ^m^ [110], ^n^ [111], ^o^ [16,79,80], ^p^ [83,112], ^q^ [93,97,113], ^r^ [77,78,114], ^s^ [110,111,115], ^t^ [19], ^u^ [116], ^v^ [86], ^w^ [32,33,34,35,36,37,38], ^x^ [117], ^y^ [84], ^z^ [87].

## Data Availability

The data presented in this study are available in the article.
